# Cancer risk after renal transplantation in South Korea: a nationwide population-based study

**DOI:** 10.1186/s12882-018-1110-3

**Published:** 2018-11-06

**Authors:** Jaesung Heo, O Kyu Noh, Young-Taek Oh, Mison Chun, Logyoung Kim

**Affiliations:** 1 Department of Radiation Oncology, Ajou University School of Medicine, Suwon, South Korea; 2 Department of Biomedical Informatics, Ajou University School of Medicine, Suwon, South Korea; 3 Office of Biostatistics, Ajou Research Institute for Innovative Medicine, Ajou University Medical Center, Suwon, South Korea; 4 Health Insurance Review and Assessment Service, Seoul, South Korea

**Keywords:** Renal transplantation, Malignancy, Screening, South Korea

## Abstract

**Background:**

This study aimed to evaluate patterns of posttransplant malignancies among renal transplant recipients (RTRs) in South Korea using nationwide data.

**Methods:**

The nationwide cohort assessed in this study included RTRs from January 1, 2010, to December 31, 2014. We analyzed cancer incidence during the time course after renal transplantation. Additionally, we calculated standardized incidence ratios (SIRs) to evaluate the risk of malignancies in RTRs.

**Results:**

A total of 1343 RTRs (871 males and 472 females, mean age 48.5 ± 11.6 years) were assessed. Among them, 104 (7.7%) developed malignancies after transplantation, most commonly in the thyroid cancer (23.1%). The SIR for all cancers was 3.54; particularly, the SIRs for renal cancer, myeloma, and non-Hodgkin lymphoma were 16.31, 24.02, and 28.64, respectively. Females showed a higher risk of malignancy than males (SIRs: 4.04 for women and 3.26 for men). The median interval between transplantation and malignancy diagnosis was 27.2 months (range 12.3–54.8 months).

**Conclusions:**

RTRs in South Korea demonstrated a high risk of malignancy after transplantation compared with the general population. This indicates that close surveillance and routine screening for cancer in RTRs are needed.

## Background

Renal transplantation (RT) is considered as the most desired treatment option for patients with irreversible chronic kidney disease [1]. There has been a measurable difference in long-term prognosis and quality of life among patients receiving transplantation compared with other treatment groups [2]. However, renal transplant recipients (RTRs) have been shown to have a higher cancer risk than the general population [3], and posttransplant malignancy is one of the major prognostic factors of mortality among RTRs [4, 5]. This is partly attributed to immunosuppression-associated malignancies in patients receiving RT [6, 7].

In Western countries, nonmelanoma skin cancer is the most common malignancy after RT [8, 9]. Meanwhile, stomach cancer, renal cancer, and transitional cell carcinoma were reported as common malignancies in Japan and China [10–12]. These findings suggest that the incidence of posttransplant malignancies varies by geographic location and ethnic population.

In South Korea, a single-center study reported malignant lymphoma and stomach cancer as the two most common types of malignancies [13]. However, the sample size of that study was insufficient to represent the nationwide incidence of malignancies after RT. Therefore, a large population study is required to assess the risk of posttransplant malignancies after RT. In the present study, we aimed to analyze cancer risk among RTRs based on a large population cohort, using claims data from the Health Insurance Review and Assessment Service (HIRA) in South Korea.

## Methods

The National Health Insurance (NHI) system is a public medical insurance system in South Korea. The HIRA database includes demographic data on sex, age, and residential areas and clinical data on diagnosis, procedures, and prescriptions. The nationwide cohort assessed in this study was based on HIRA claims data and included patients who received RT from January 1, 2010, to December 31, 2014. This study was approved by the institutional review board (IRB) of Ajou University Hospital (IRB No. AJIRB-MED-EXP-16-493).

Patients were identified by procedure codes indicating RT (R3280). Moreover, we confirmed use of immunosuppressants such as azathioprine, cyclosporine, prednisolone, mycophenolate mofetil, tacrolimus, and sirolimus. Malignancies after RT were confirmed using the main disease codes in claims data from inpatient and outpatient first visits. Malignancies were diagnosed based on pathological findings and identified by principal diagnoses using codes based on the International Classification of Diseases, 10th Revision. We excluded malignancies diagnosed before RT as well as in situ malignancies.

We analyzed the patterns of malignancies during the time course after RT. Descriptive statistics were calculated to estimate the frequency of the cancer site using the diagnostic code based on the first hospital visit. We also analyzed standardized incidence ratios (SIRs) and corresponding 95% confidence intervals (95% CIs) to estimate the relative risk of malignancies between RTRs and the general population [14]. All statistical analyses were performed with R 3.0.2.

## Results

**Fig. 1:**
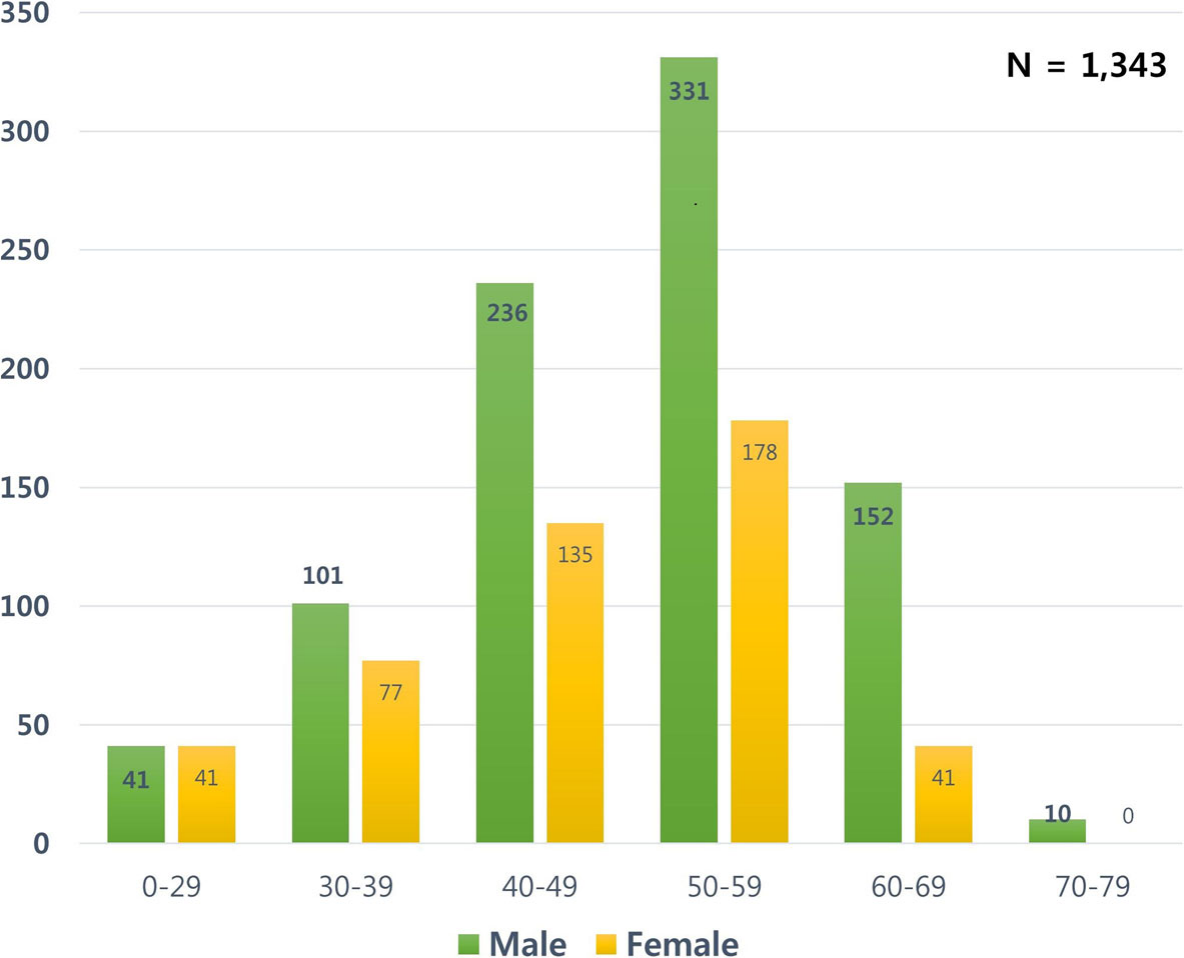
Overall distribution of patients with renal transplantation according to age and sex

A total of 1343 patients who received RT between 2010 and 2014 were identified in South Korea. The median age of these patients was 50 years (range 1–75 years); 871 patients (64.9%) were men and 472 (35.1%) were women (Fig. 1). Although the total percentage of males was high, the percentage of females was relatively high in the age group of < 30 years. Of the 1343 patients, 104 (7.7%) had malignancies during the follow-up period.

The mean follow-up time for RTRs was 25.4 ± 16.7 months. The most common malignancies after RT were thyroid cancer (23.1%), renal cancer (8.7%), stomach cancer (6.7%), liver cancer (5.8%), lung cancer (5.8%), and breast cancer (5.8%) (Table 1). Kaposi’s sarcoma (*n* = 3), stomach cancer (*n* = 7), pancreatic cancer (*n* = 2), and larynx cancer (*n* = 1) were confirmed only in males. The cancers that were found only in females were breast cancer (*n* = 7), gynecologic cancer (*n* = 3), colon cancer (*n* = 1), anal cancer (*n* = 1), and ureteral cancer (*n* = 1).

**Table 1:** Types of post-transplantation malignancy from 2010 to 2014 in South Korea

All cancer	Total (%)	Male, N (%)	Female, N (%)
	104 (100)	63 (100)	41 (100)
Thyroid (C73)	24 (23.1)	12 (19.0)	12 (29.3)
Kidney (C64)	9 (8.7)	7 (11.1)	2 (4.9)
Stomach (C16)	7 (6.7)	7 (11.1)	
Liver (C22)	6 (5.8)	5 (7.9)	1 (2.4)
Lung (C34)	6 (5.8)	5 (7.9)	1 (2.4)
Breast (C50)	6 (5.8)		6 (14.6)
Leukemia (C91, C92)	4 (3.8)	2 (3.1)	2 (4.9)
Skin (C44)	4 (3.8)	3 (4.8)	1 (2.4)
Myeloma (C90)	4 (3.8)	3 (4.8)	1 (2.4)
Non-Hodgkin lymphoma (C83, C85)	4 (3.8)	3 (4.8)	1 (2.4)
Kaposi sarcoma (C46)	3 (2.9)	3 (4.8)	
Cervix uteri (C53)	3 (2.9)		3 (7.3)
Prostate (C61)	3 (2.9)	3 (4.8)	
Brain (C71)	3 (2.9)	1 (1.6)	2 (4.9)
Pancreas (C25)	2 (1.9)	2 (3.1)	
Bladder (C67)	2 (1.9)	1 (1.6)	1 (2.4)
Rectum (C19, C20)	2 (1.9)	1 (1.6)	1 (2.4)
Parotid gland (C07)	1 (0.1)	1 (1.6)	
Colon (C18)	1 (0.1)		1 (2.4)
Anus (C21)	1 (0.1)		1 (2.4)
Larynx (C32)	1 (0.1)	1 (1.6)	
Corpus uteri (C54)	1 (0.1)		1 (2.4)
Ovary (C56)	1 (0.1)		1 (2.4)
Renal pelvis (C65)	1 (0.1)		1 (2.4)
Ureter (C66)	1 (0.1)		1 (2.4)
Other site	4 (3.8)	3 (4.8)	1 (2.4)

The median age at diagnosis of malignancy was 52 years (range 2–67 years). Patients with leukemia had a median age of 32 years, whereas those with prostate cancer had a median age of 67 years (Table 2). The median interval between RT and diagnosis of malignancy was 27.2 months (range 12.3–54.8 months). Leukemia, liver cancer, stomach cancer, lymphoma, and prostate cancer occurred relatively earlier. In contrast, lung cancer, breast cancer, and thyroid cancer were confirmed at a relatively later time.

**Table 2:** Time from renal transplant to cancer diagnosis and median age at diagnosis

All cancers	Age (years)		Interval (months)	
	median	range	median	range
	52	2–67	27.2	12.3–54.8
Thyroid	46	28–63	31.6	14.5–54.8
Kidney	42	37–59	26.3	12.3–44.5
Stomach	58	50–66	22.9	13.9–40.4
Liver	57	46–62	22.2	12.3–32.5
Lung	60	57–67	34.2	18.9–54.0
Breast	47	38–53	32.5	20.5–47.8
Leukemia	32	2–52	16.6	12.6–34.6
Skin	61	48–67	26.6	19.8–31.4
Myeloma	50	39–64	26.9	22.0–47.6
Non-Hodgkin lymphoma	41	26–69	24	18.7–33.0
Kaposi sarcoma	60	47–65	33.2	13.6–37.2
Cervix uteri	54	35–61	27	14.6–52.5
Prostate	67	61–67	25.5	14.4–26.6
Brain	62	46–63	19	14.1–49.2
Pancreas	64	60–67	34.8	24.7–44.9
Bladder	53	49–57	21.1	12.3–29.8
Rectum	50	50	28.9	14.1–43.6
Thyroid	52	2–67	27.2	12.3–54.8

Overall cancer risk was significantly increased in RTRs compared with the general population (SIR 3.54, 95% CI 2.89–4.29; Table 3). Overall, the SIRs for patients with thyroid cancer, renal cancer, liver cancer, breast cancer, leukemia, skin cancer, myeloma, non-Hodgkin lymphoma, Kaposi’s sarcoma, cervical cancer, brain tumor, and bladder cancer were significantly higher than those of the general population. Renal cancer (SIR 16.31), myeloma (SIR 24.02), non-Hodgkin lymphoma (SIR 28.64), and leukemia (SIR 27.08) demonstrated significantly higher SIRs than all other cancer types.

**Table 3:** Standardized incidence ratios for different types of cancer developed after renal transplantation from 2010 to 2014 in South Korea

All cancer	Total patients				Males				Females			
	Obs.	Exp.	SIR	95% CI	Obs.	Exp.	SIR	95% CI	Obs.	Exp.	SIR	95% CI
	104	29.35	3.54	2.89–4.29*	63	19.32	3.26	2.51–4.17*	41	10.16	4.04	2.90–5.47*
Thyroid	24	5.24	4.58	2.94–6.82*	12	1.25	9.57	4.94–16.71*	12	3.00	4.00	2.06–6.98*
Kidney	9	0.55	16.31	7.44–30.95*	7	0.49	14.15	5.67–29.15*	2	0.12	16.68	1.87–60.23*
Stomach	7	4.11	1.71	0.68–3.51	7	3.58	1.95	0.78–4.03				
Liver	6	2.19	2.74	1.00–5.97*	5	2.11	2.37	0.76–5.53	1	0.39	2.54	0.03–14.13
Lung	6	3.34	1.79	0.66–3.91	5	2.73	1.82	0.59–4.27	1	0.64	1.56	0.02–8.66
Breast	6	2.22	2.70	0.99–5.88*					6	1.56	3.85	1.41–8.39*
Leukemia	4	0.15	27.08	7.28–69.32*	2	0.26	7.78	0.87–28.10*	2	0.10	19.26	2.16–69.54*
Skin	4	0.53	7.58	2.04–19.40*	3	0.29	10.41	2.09–30.40*	1	0.21	4.65	0.06–25.91
Myeloma	4	0.17	24.02	6.46–61.50*	3	0.12	25.90	5.20–75.67*	1	0.05	18.92	0.24–105.25
Non-Hodgkin lymphoma	4	0.14	28.64	7.70–73.32*	3	0.10	30.21	6.07–88.28*	1	0.19	5.21	0.07–28.96
Kaposi sarcoma	3	0.01	446.76	89.79–1305.35*	3	0.08	37.85.	7.61–110.59*				
Cervix uteri	3	0.50	6.05	1.22–17.69*					3	0.18	16.73	3.36–48.87*
Prostate	3	1.22	2.45	0.49–7.16	3	0.86	3.49	0.70–10.19				
Brain	3	0.23	12.91	2.60–37.73*	1	0.16	6.17	0.08–34.34	2	0.08	26.16	2.94–94.44*
Pancreas	2	0.72	2.79	0.31–10.07	2	0.50	3.97	0.45–14.34				
Bladder	2	0.49	4.06	0.46–14.65*	1	0.51	1.95	0.03–10.85	1	0.07	14.61	0.19–81.30
Rectum	2	1.64	1.22	0.13–4.41	1	1.34	0.97	0.01–4.15	1	0.42	2.35	0.03–13.10
Corpus uteri	1	0.50	2.02	0.03–11.23					1	0.19	5.23	0.07–29.11
Ovary	1	0.29	3.40	0.04–18.92					1	0.21	4.86	0.06–27.04
Colon	1	2.08	0.48	0.01–2.67					1	0.61	1.63	0.02–9.09

*p value < 0.05

Female RTRs showed a higher risk for the development of all cancers than male RTRs (females: 4.04, 95% CI 2.90–5.47; males: 3.26, 95% CI 2.51–4.17). Among all cancer types, brain tumor (SIR 26.16), leukemia (SIR 19.26), renal cancer (SIR 16.68), and cervical cancer (SIR 16.73) represented the highest risk of malignancy in females. Meanwhile, males had a high risk for developing myeloma (SIR 25.90), non-Hodgkin lymphoma (SIR 30.21), and Kaposi’s sarcoma (SIR 37.85).

## Discussion

The long-term use of immunosuppressants and dialysis therapy before RT has been shown to increase the risk of malignancies in RTRs compared with the general population [15–17]. The development of techniques for transplantation can also affect the incidence of malignancies. Recently, the risks of graft failure and death after RT have been reduced over time, with improved survival; however, increased survival time could contribute to more chances of developing a second primary malignancy [18, 19]. In our study, cancer occurred in 104 patients (7.7%), comprising both sexes, and cancer risk was significantly increased in RTRs compared with the general population (SIR 3.54, 95% CI 2.89–4.29).

Another study in South Korea reported a 4.3% rate of malignant development during a 37-year follow-up after RT at a single center [20]. Kim et al. also confirmed a 4.2% rate of malignancy after RT among 757 patients [21]. In a study by Choi et al., malignancies after RT were found in 7.2% of patients [13]. Moreover, another study based on the Taiwanese population found 320 cases (6.8%) of posttransplant malignancies in 4716 RTRs [12]. Such finding is similar or slightly higher than those of previous studies, which could be because the subjects in the study were limited to patients who recently underwent transplantation between 2010 and 2014. Compared with past studies, the study efficiently conducted intensive follow-up after transplantation and performed advanced examination techniques for screening. Therefore, the risk of malignancy after RT could vary according to the study period of RT [16].

In our study, the distribution of sites of posttransplant malignancies differed from that of studies in Western populations. Skin cancer is the most common malignancy in Western countries. However, the incidence of skin cancer is low in Asian countries. In Japan, stomach cancer, renal cancer, and lymphoma are more common [10, 11], whereas in Taiwan, renal, bladder, and liver cancer are the most common malignancies [12]. In our study, thyroid cancer (23.1%), renal cancer (8.7%), stomach cancer (6.7%), and liver cancer (5.8%) were the most common cancers. Thyroid cancer was appreciably higher in our results, and such high incidence in South Korea is considered to be due to geographical characteristics [14, 22, 23]. Skin cancer was identified in only four patients (3.8%), of whom three were male, which is consistent with the results in other Asian countries [24]. However, the risk of developing Kaposi’s sarcoma was highest among all malignancies (SIR 446.76). Meanwhile, the risk of developing lymphoma was also high in our study, which confirms the results of a previous meta-analysis for an Eastern country [16].

The age at diagnosis of malignancy was different between cancer types (Table 2). Thyroid cancer, breast cancer, leukemia, renal cancer, and non-Hodgkin lymphoma were diagnosed at a relatively low age of < 50 years. However, skin cancer, prostate cancer, pancreatic cancer, and brain tumors were confirmed in elderly patients aged > 60 years. Compared with Western countries, in South Korea, breast cancer is more prevalent at a young age; thus, local epidemiologic characteristics should also be considered [25]. Furthermore, in our study, the interval between RT and diagnosis of cancer also varied according to cancer type. With regard to sex, cancer risk was higher in women than in men (SIR 4.04 vs 3.26) [16]. Therefore, it is important to consider a screening for malignancies that is tailored to age, sex, and cancer type.

In our study, thyroid cancer, gynecologic cancer, prostate cancer, and lymphoma were confirmed in 36 patients. These cancer types are difficult to detect using only abdominal and chest computed tomography (CT). Particularly, cervical cancer detection requires Pap smears annually. Our study also confirmed three patients who were diagnosed with colorectal cancer after RT. Although fecal occult blood tests for colorectal screening have a low false-negative rate, flexible sigmoidoscopy for pathological confirmation is ultimately needed. Therefore, a screening test is needed for identifying these difficult-to-detect cancers. Promoting health examinations among RTRs and using 18F-fluorodeoxyglucose (FDG) positron emission tomography (PET)-CT in evaluating status in a systematic manner could be considered. FDG PET-CT has not been useful in detecting primary prostate cancer. However, it can be useful for detecting posttransplant malignancies as a systematic screening test, although its cost-effectiveness may be a concern. A previous study has confirmed that appropriate screening and early detection of cancer could reduce mortality and improve prognosis of RTRs [5].

Besides screening, lifestyle modifications are also important. Lung cancer was confirmed in six patients (five males, one female) in this study. Since lung cancer is associated with smoking, RTRs should be strongly encouraged to quit smoking, and screening with low-dose CT is recommended for smokers. On the other hand, six patients were diagnosed with liver cancer (SIR 2.74). South Korea is within the epidemic region of hepatitis B, which is an important risk factor of liver cancer. Therefore, active attention is necessary in the management of viral infections through the use of vaccination among RTRs. Before transplantation, patients should receive vaccination for hepatitis B virus if they do not already have the specific antibody.

This study has several limitations. The insurance claims registry was limited in terms of accuracy and validity and due to absence of universal screening methods. Clinical data such as type of pathology, laboratory data, and imaging results were underreported. Moreover, the data lacked social factors associated with cancer risk, such as lifestyle (smoking, alcohol use) and family history. Since the period of legal use of HIRA claims data is limited to 5 years, the shorter follow-up period in this study relative to that in previous studies was not adequate to detect malignancies with a late onset of ≥5 years. Therefore, further studies with a longer follow-up period should be conducted.

## Conclusions

This study confirmed that cancer risk was significantly increased in RTRs compared with the general population. The types and risk of cancer after RT in South Korea were different from those in other countries. This geographical variation would be helpful in improving cancer screening and follow-up recommendations for RTRs in Asian countries, specifically in South Korea.

## Abbreviations

CI: Confidence interval
CT: Computed tomography
FDG: 18F-fluorodeoxyglucose
PET: Positron emission tomography
RT: Renal transplantation
RTR: Renal transplant recipients
SIR: Standardized incidence ratios
